# Design of Fermentative Technology for the Valorization of Pig Bristle Keratins into Biostimulant for Agricultural Applications

**DOI:** 10.1021/acsagscitech.5c00324

**Published:** 2025-09-16

**Authors:** Angel Orts, Jesús López, José M. Orts, Salvadora Navarro-Torres, Emilia Naranjo, Pablo Caballero, Luis Martín-Presas, Angélica Castaño, Juan Parrado

**Affiliations:** † Departamento de Bioquímica y Biología Molecular. Facultad de Farmacia, 16778 Universidad de Sevilla, C/Prof. García González 2, Sevilla 41012, Spain; ‡ Departamento de Microbiología y Parasitología, Facultad de Farmacia, 16778 Universidad de Sevilla, Sevilla 41012, Spain

**Keywords:** keratins, pig bristles, fermentation, biostimulant

## Abstract

The elimination of keratin-derived waste, such as pig bristles, represents a significant challenge due to its high production levels and resistance to degradation. However, the keratinous composition also makes pig bristles a valuable waste material with significant potential for bioconversion into biostimulants rich in bioavailable nitrogen, peptides, and amino acids. To achieve degradation, microorganisms with keratinolytic activity isolated from the raw material were selected. Based on the best performance in plant PGP traits, solubility, and protease activity, *Sporosarcina luteola* was chosen to implement a fermentation technology that converts pig bristle waste. The fermented product comprises three classes of biostimulant components: the biomass of *S. luteola*, the enzymatic secretions of this microorganism, and the hydrolyzed organic matter from pig bristles, which is rich in protein hydrolysates and free amino acids. The biostimulant was evaluated in soil at the biochemical level (enzymatic activities) and in plants under oxidative stress, demonstrating a positive effect. These findings highlight the fermentation process using *S. luteola* as a promising strategy for the comprehensive valorization of pig bristle waste, resulting in products with significant agronomic and environmental benefits.

## Graphical abstract

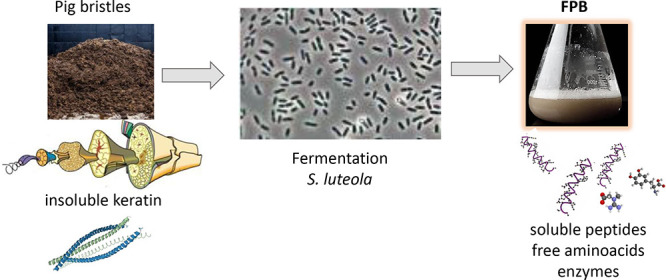

## 1 Introduction

The constant growth of the population leads to the generation of vast amounts of waste from human food consumption, creating an environmental issue that urgently needs to be addressed globally. In this context, the European Union has recognized the development of a bioeconomy as a priority, emphasizing the recycling of waste from diverse sources to minimize environmental impact.^1^ Among these waste materials, byproducts derived from pigs, such as bristles, have a significant environmental impact. According to Eurostat data,^2^ approximately 134 million head pigs were slaughtered in the European Union in 2022. Since each slaughtered pig produces around 0.9 kg of bristles, this results in approximately 120.000 tons of pig bristle waste annually, contributing to pollution due to the lack of proper management.

However, these discarded materials have the potential to serve as raw resources for generating valuable chemicals applicable in various sectors, such as biofertilizers or biostimulants for agriculture.^3,4^ Like other vertebrate skin appendages with a protective function, such as hair, feathers, or nails, pig bristles are composed of keratins. These are proteins characterized by a three-dimensional fibrillar structure rich in sulfur, also known as hard keratins.^5,6^ Such keratinous materials have a high protein content comprising approximately 40% hydrophilic and 60% hydrophobic amino acids. The degradation of keratin waste can therefore provide an inexpensive source of digestible proteins and amino acids, which can be utilized in applications such as animal feed or fertilizers. These materials are difficult to digest due to their structural complexity, which is based on a two-phase organization where tightly packed, extensively cross-linked polypeptide chains are embedded in an amorphous high-cysteine protein matrix.^6^ This structure gives them unique resilience to mechanical stress and resistance to cleavage by common proteases, making extensive pretreatment necessary to obtain usable products. Various physicochemical methods have been applied for this purpose.^7^ However, these methods come with both economic and environmental costs that must be considered. As a result, there is growing interest in biological keratin waste degradation, which is emerging as a more cost-effective and environmentally friendly alternative to chemical and hydrothermal methods. Biological degradation, utilizing keratinophilic microorganisms or their enzymes (keratinases), offers an ecofriendly solution for the hydrolysis and recycling of keratin waste. This approach not only increases the commercial value of keratin waste but also operates under milder conditions, facilitating the production of valuable byproducts.^8^

Our research group has previously developed microbiological and enzymatic technologies to obtain biofertilizers and biostimulants from agro-industrial residues, both from plant sources, such as okara^9–11^ and rice bran,^12^ and from animal sources, such as chicken feathers, and even from sewage sludge.^13,14,16,17^ Our technology involves the controlled application of enzymes or the use of fermentation processes to obtain high-quality extracts rich in peptides and bioactive compounds that have demonstrated positive agronomic effects, such as improving nutrient absorption, regulating and increasing tolerance to abiotic stress, reducing the need for conventional fertilizers, and stimulating microbial communities in the soil. All of this makes them suitable to be considered a biostimulant according to its definition.^18^

Keratin-degrading bacteria play a crucial role in nutrient cycling and improving soil quality and have gained attention in agriculture due to their ability to break down keratinous proteins found in organic waste,^19^ converting them into peptides and amino acids with high potential as nitrogen sources for soils and plants.^20^ The use of keratinous waste hydrolysates derived from fermentations with keratin-degrading bacteria has shown positive effects on soil quality, highlighting the proliferation of beneficial microorganisms, improving soil coverage, and promoting plant growth, quality, and protection against environmental stressors.^21^

Given the above, the aim of this study was to assess the biostimulant potential of protein-rich extracts obtained from the fermentation of pig bristles. For this purpose, agro-industrial pig bristle waste was subjected to a fermentation process using bacteria isolated from the keratinous material itself. The fermented extract with the highest solubilization rate, protease activity, and plant growth-promoting (PGP) traits was selected to assess its biostimulation capacity both in soil, by evaluating changes in biochemical parameters, and in pepper plants subjected to ozone-induced stress.

## 2 Materials and Methods

### 2.1 Analysis of the Chemical Composition

The chemical composition of pig bristles and the soluble fermented extract was analyzed by the Microanalysis Service of the University of Seville (CITIUS, US). The amino acid composition, both free and total amino acids, was determined by FITOSOIL (Seville, Spain).

### 2.2 Isolation and Characterization of Pig Bristle Bacteria

#### 2.2.1 Bacterial Identification by 16S rRNA Amplification

Discarded pig hair waste supplied by Tara (Calasparra, Murcia, Spain) was mixed with a 0.9% sterile saline solution for 10 min. Subsequently, this suspension was plated on a mineral salt solid medium^22^ with a pH of 7.2 and cultivated at 37 °C. Three bacteria with bacillus morphology were isolated.

For their identification, the isolated bacteria were stored on Indicating FTATM Micro Card according to the manufacturer’s instructions and were sent to STAB Vida (Lisboa, Portugal).

The extraction and purification of the input DNA were performed by STAB Vida (Lisboa, Portugal) following the 16S rRNA identification method. The 16S rRNA gene partial sequence was deposited in the GenBank/EMBL/DDBJ database with its corresponding accession number (complete 16S rRNA gene sequence can be found under accession numbers PQ877673, PQ877694, PQ877697).

#### 2.2.2 PGP Properties

The isolated bacteria were analyzed for multifarious PGP mechanisms such as indole acetic acid-producing ability,^23^ siderophore production,^24^ and solubilization of inorganic phosphate.^25^ Enzymatic activity, 1-aminocyclopropane-1-carboxylate (ACC) deaminase, of PGPB was determined as described by^26^. The total protein content of bacterial cells was determined by the Bradford reagent protocol.^27^ Final ACC deaminase activity was expressed in nanomoles of α-ketobutyrate mg^–1^ protein h^–1^.

Biofilm formation and nitrogen fixation were also evaluated. To evaluate biofilm formation, all bacterial strains were individually cultured in 24-well plates containing the TSB medium for 4 days at 28 °C. After the incubation period, the position of the biofilm within the wells (surface or bottom) was assessed. Biofilms were then stained with 0.01% crystal violet (w/v) following the method described by Del Castillo et al.^28^ to determine whether bacterial biomass adhered to the walls of the wells, indicating ring formation.

To assess the ability of bacteria to fix atmospheric nitrogen, they were incubated at 28 °C for 5 days on a nitrogen-free broth solid medium, as described by Ji et al.^29^ Bacterial strains capable of growing on this medium were identified as nitrogen-fixing.

Finally, production of auxins was determined according to the standard method of ref 30.

#### 2.2.3 Enzymatic Activities

To characterize isolated bacteria, several enzymatic activities were analyzed. Pectinase and cellulase activities were examined according to the method previously described.^31^ Amylase activity was performed in starch agar plates incubated for 7 days at 28 °C and revealed with 10 mL of lugol. Lipase and protease activities were observed by the presence of halos around bacteria after incubation in Tween and casein agars, respectively, for 7 days at 28 °C.^32^ Concerning DNAsa activity, bacteria were incubated for 7 days at 28 °C in DNA agar plates. The plates were then developed with 1 M HCl. Halos in the dark background were observed in bacteria with DNAsa activity. Chitinase activity was performed as described in^33^.

### 2.3 Fermentative Processes

The fermentation process was carried out in 500 mL Erlenmeyer flasks under controlled agitation and temperature conditions, using bacteria isolated from pig bristles. These microorganisms were stored frozen at −80 °C and thawed 24 h before inoculation in the LB medium.

The culture medium was prepared in advance, containing pig bristles at concentrations of 2, 5, and 10% (w/v). A liquid mineral salt medium was used.^22^ The initial pH was 6.81, and the media were sterilized at 121 °C and 1 atm for 30 min. After sterilization, a suspension of the isolated bacteria was added at a concentration of 2% (w/v). The fermentation conditions were set to a temperature of 37 °C with agitation at 120 rpm and an operating time of 240 h. The samples were then centrifuged for 40 min at 4 °C and 10,000 *g* (Avanti J-26XP, Beckman-Coulter).

#### 2.3.1 Solubility

To assess solubility, the soluble fractions of fermented pig bristles were heat-dried and analyzed, while the pellets were weighed and discarded. As negative controls, the same extractions were performed without bacteria using only water (soluble control).

#### 2.3.2 Protease Activity

Total extracellular protease activity was determined as previously described.^34^ Briefly, 0.5 mL of azocasein 1% (w/v) in 0.1 M phosphate buffer (pH 7) was mixed with 0.5 mL of the sample. This was incubated for 10 min at 40 °C. The reaction was terminated by adding 2.5 mL of a 5% (p/V) TCA solution. The reaction mixture was centrifuged, and the absorbance of the supernatant at 440 nm was measured. One unit of proteolytic activity was defined as the amount of enzyme required to produce an increase in the optical density of 0.001.

#### 2.3.3 Analysis of the Molecular Weight of Soluble Proteins

The molecular mass distribution of protein in the samples was determined by size-exclusion chromatography using a Jasco UV4075, Superdex 30 increase 10/300GL column. Proteins/peptides were detected at 280 and 215 nm with a JASCO UV-4075 UV/vis detector module coupled to the column. The operational conditions have been described in a previous study.^35^

### 2.4 Study on Biostimulant Properties of the Soluble Fraction of Fermented Pig Bristles

#### 2.4.1 Soil Biostimulation Study

The experimental design was established according to a previous study.^11^ Briefly, microcosms of 250 g of soil were preincubated at 30–40% of their water-holding capacity for 7 days. After this phase, each product was added to the soil. Soil without the addition of any product was used as the control (SC group), while the experimental groups consisted of soil with the addition of raw pig bristle (SPB group) and the experimental SFPB group consistent in soil treated with the soluble fraction of fermented pig bristles from bacteria selected based on the best performance in plant PGP traits, solubility, and protease activity.

Each product was evaluated at 1% w/w (dry matter). After 1, 3, 7, 10, 15, 30, and 55 days of the incubation period and for each treatment, the dehydrogenase, phosphatase, and β-glucosidase enzymatic activities were determined using the methods described in^36^ and^37^.

#### 2.4.2 Study in Plants

##### 2.4.2.1 Plant Treatment

To analyze the biostimulant potential and defense against environmental stress caused by ozone, the soluble fraction of the selected fermented pig bristles (hereafter referred to as FPB groups) was applied to pepper plants. Treatments were applied according to a previous work by our group. Briefly, *Capsicum annum* L. var. *grossum* (pepper) plants were raised from seeds in plastic pots and grown inside the University of Seville Glasshouse General Services following a protocol previously described. After 8 days of transplantation, 20 pepper plants were selected and divided into four groups and were foliar-sprayed a total of 4 times at 5 day intervals, with an aqueous solution of selected FPB at 1% (w/v) (groups FPB and FPB + O_3_) or distilled water (groups Ct and Ct + O_3_). After 5 days of the last spray treatment, Ct + O_3_ and FPB + O_3_ plants were transferred to a phytoclimatic chamber with an ozone generator (ZONOSISTEM GM 5000 O_3_ Generator) attached and exposed to three consecutive fumigations with 100 ppb of O_3_ for 6 h (from 10:00 a.m. to 4:00 p.m.).

After ozone fumigation, all of the test plants were sprayed again with the corresponding solution (PBF 1% or distilled water). Finally, 24 h after the last exposure to ozone, foliar samples were taken from each plant, and the analyses described below were carried out.

##### 2.4.2.2 Plant Status after Ozone Exposition

*Physiological Status in Plants*

To evaluate the physiological state of the plants, various photosynthetic parameters such as net photosynthetic rate (*A*_N_), electron transport rate (ETR), and effective quantum yield of photosystem II (PhiPSII) were analyzed using an IRGA (LI-6400XT, LICOR Inc., Nev., EEUU) with a light chamber for the leaf (Li-6400–02B, Li-Cor Inc.) according to ref 12.

Additionally, delayed fluorescence measurements were also detected using a plant imaging system (NightShade LB 985, Berthold Technologies, Germany) equipped with a deeply cooled CCD camera according to ref 38.

##### 2.4.2.3 Oxidative Stress Evaluation in Plants

To evaluate the oxidative stress of plants, antioxidant enzymatic activities and MDA were analyzed.

##### 2.4.2.4 Antioxidant Enzymes

Enzymatic activities of ascorbate peroxidase (APX), superoxide dismutase (SOD), guaiacol peroxidase (GPX), and catalase (CAT) were measured as described by Duarte et al.^39^ Briefly, vegetal extract was extracted in an extraction buffer (50 mM sodium phosphate buffer; pH 7.6) from 500 mg of leaves. CAT activity was determined at 240 nm in a reaction solution containing an assay buffer (50 mM sodium phosphate buffer, pH 7.0) and 100 mM H_2_O_2_. APX activity was assayed in the assay buffer with 12 mM H_2_O_2_ and 0.25 mM L-ascorbate and measured at 290 nm. SOD activity was determined by monitoring the pyrogallol oxidation at 325 nm by the addition of 3 mM pyrogallol. GPX activity was measured at 470 nm in a reaction mixture containing the assay buffer, 2 mM H_2_O_2_, and 20 mM guaiacol. To determine the auto-oxidation of the substrates, control assays were performed in the absence of enzymatic extract samples.^39^

##### 2.4.2.5 MDA

Ozone-induced oxidative stress in pepper plants was analyzed through lipid peroxidation. For that, malondialdehyde (MDA) content was determined in leaf homogenates, using the thiobarbituric acid reactive substances assay.^40^

### 2.5 Statistical Analysis

Statistical analysis was conducted using GraphPad Prism 8.4.0.671. Normality was assessed using the Kolmogorov–Smirnov test. The means of the different treatments were compared using a two-way ANOVA, and statistical differences were determined using the Tukey multiple comparison test.

## 3 Results

### 3.1 Pig Bristle Characterization

A prior characterization of the raw material was conducted. As shown in Table 1, the raw pig bristles contained 93.0% dry matter, of which the protein fraction (including both soluble and insoluble proteins) represented 93.7% (N 16.4% × 5.7). The complete amino acid composition of the pig bristles was further analyzed and is also presented in Table 1. The amino acid composition shows a rich variety of all amino acid groups, with a notably high content of glutamic acid (19.8%).

**Table 1:** Chemical Composition and Amino Acid Content of Pig Bristles and Soluble Fraction of Fermented Pig Bristles with *S. luteola* (FPB)

	pig bristles	FPB
(% w/w of dry matter)		
dry matter	93.0 ± 0.5	88.2 ± 0.3
humidity	6.9 ± 0.5	11.8 ± 0.3
soluble fraction	10.0 ± 0.2	100 ± 0.1
insoluble fraction	90.0 ± 0.2	0 ± 0.1
ash	3.4 ± 0.1	3.4 ± 0.1
organic material	96.6 ± 0.4	86.4 ± 0.1
C	56.0 ± 0.2	50.1 ± 0.3
N	16.4 ± 0.1	15.62 ± 0.2
pH	5.9	8.2

(%)		
glutamic acid	19.8 ± 1.5	25.1 ± 1.8
arginine	9.1 ± 1.1	8.3 ± 0.9
aspartic acid	9.1 ± 0.9	8.4 ± 0.9
leucine	8.9 ± 0.8	8.4 ± 0.8
serine	7.4 ± 0.5	6.8 ± 0.5
proline	6.8 ± 0.5	6.4 ± 0.5
valine	6.5 ± 0.6	6.2 ± 0.4
alanine	5.3 ± 0.2	5.5 ± 0.4
glycine	5.2 ± 0.4	5.3 ± 0.3
threonine	5.2 ± 0.5	4.1 ± 0.3
lysine	4.2 ± 0.2	3.8 ± 0.4
tyrosine	3.8 ± 0.4	3.6 ± 0.3
isoleucine	3.6 ± 0.3	3.6 ± 0.3
phenylalanine	2.7 ± 0.2	2.3 ± 0.2
methionine	0.9 ± 0.1	1.0 ± 0.1
histidine	0.9 ± 0.1	0.6 ± 0.1

### 3.2 Isolation and Characterization of Pig Bristle Bacteria

From the raw pig bristles, three bacterial strains with bacillus morphology were found, named ORT1 (PQ877673), ORT2 (PQ877694), and ORT3 (PQ877697). The results of the 16S rRNA gene sequencing show that the closest species according to the NCBI database were *Bacillus licheniformis* , *Sporosarcina luteola,* and *Bacillus fordii*, respectively (Table 2). The ORT2 and ORT3 strains showed an identity percentage lower than 100%, suggesting that they could be new species.

**Table 2:** Identification of the Bacteria Isolated from Pig Bristles

strain	bacterial species	identity (%)
ORT1 (PQ877673)	*Bacillus licheniformis*	100
ORT2 (PQ877694)	*Sporosarcina luteola*	98.60
ORT3 (PQ877697)	*Bacillus fordii*	99.85

Regarding the PGP properties, all of the isolated bacteria exhibited at least one of the studied properties (Table 3). The three strains had the ability to form biofilms, and all of the strains solubilized siderophores, with *S. luteola* standing out with a 0.6 cm ring, and all of the strains seemed to possess the ability to fix nitrogen. However, regarding the production of auxins, none of the bacteria tested positive for this property.

**Table 3:** PGP Properties and Enzyme Activities Present in the Bacteria Isolated from Pig Bristles (±, Weak Activity)

	biofilm	auxins	siderophores (cm)	phosphates	nitrogen fixation
*Bacillus licheniformis*	bottom and ring	–	0.5	±	+
*Sporosarcina luteola*	bottom	–	0.6	±	+
*Bacillus fordii*	bottom and ring	–	0.3	–	+

	DNase	amylase	cellulase	lipase	pectinase	protease	chitinase
*Bacillus licheniformis*	+	+	+	+	+	+	+
*Sporosarcina luteola*	+	+	+	+	+	+	+
*Bacillus fordii*	+	–	–	+	–	+	–

For characterization of the isolated bacteria, the presence of several enzymatic activities, including DNase, amylase, cellulase, lipase, pectinase, protease, and chitinase, was studied (Table 3). All activities were present in *B. licheniformis* and *S. luteola*. However, *B. fordii* only exhibited expression for DNAase, lipase, and protease activities.

### 3.3 Fermentative Bioprocess

The fermentative treatment was chosen by using bacteria that had been isolated and previously sequenced to determine their potential for degrading keratins into peptides and amino acids at different concentrations of pig bristles. As observed in Figure 1A, as the concentration of keratins (pig bristles) in the medium increases, solubility decreases. Regarding the strain with the highest performance, *S. luteola* degraded 51.37% of the bristles present in the medium at a concentration of 2%, over 10 days of fermentation.

**Figure 1:**
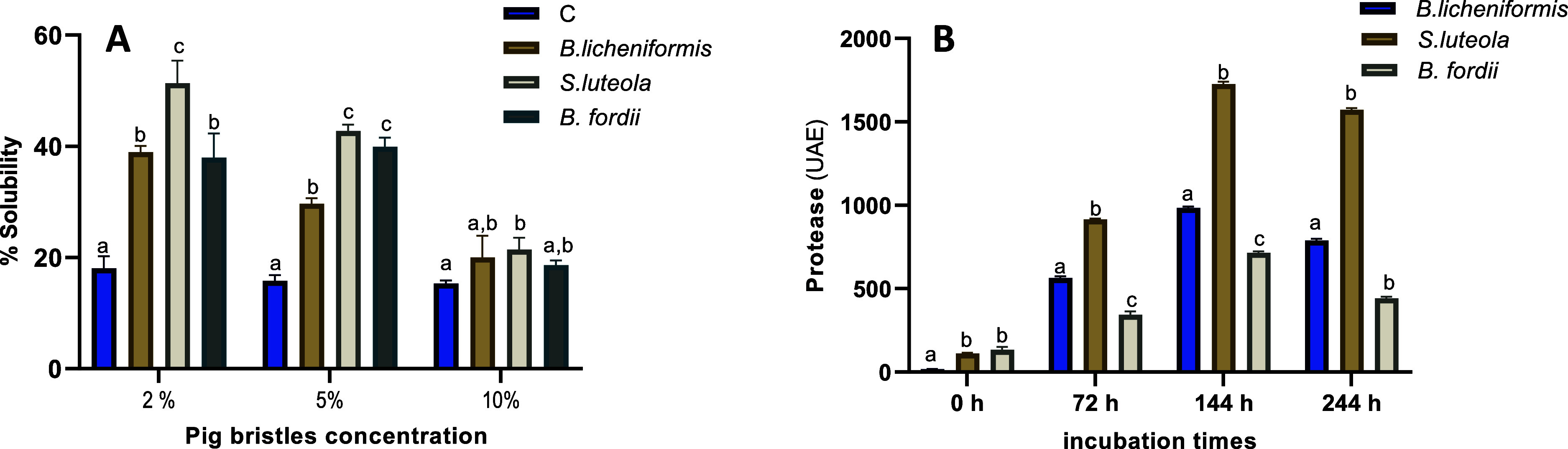
(A) Solubility of the fermented medium containing pig bristles at different concentrations (2, 5, and 10%) after 10 days of inoculation with bacteria isolated from raw material ( *B. licheniformis* , *S. luteola*, *B. fordii*). As a negative control, the same medium without bacteria was used. Control and fermented extracts were compared for each concentration of pig bristles in the medium. Values represent mean ± SD, *n* = 3. Different letters indicate means that are significantly different from each other (two-way ANOVA, HSD test, *P* < 0.05). (B) Protease activity (UAE) of the fermented media containing 2% pig bristles at different times after inoculation. Activities in fermented extracts were compared for each time point. Values represent mean ± SD, *n* = 5. Different letters indicate means that are significantly different from each other (two-way ANOVA, HSD test, *P* < 0.05).

In line with this result, protease activity was analyzed in fermented media containing 2% pig bristles. The highest values were observed in the medium fermented with *S. luteola*, with values significantly different from the other two samples at all evaluated time points (Figure 1B), reaching maximum activity at 144 h (175.6 and 241.1% compared to *B. licheniformis* and *B. fordii,* respectively).

Therefore, pig bristle fermentation with *S. luteola* was selected for the preparation of the biostimulant applied to soils and plants, with the fermentation process behaving like the normal fermentation of keratins. In addition to the visual degradation of the substrate, alkalinization of the culture media was also observed after 48 h. After centrifugation of fermented pig bristles, the soluble fraction (FPB) was separated from insoluble keratins and bacteria biomass.

### 3.4 Chemical Characterization of the Soluble Fraction of Fermented Pig Bristles

The water-soluble extract was dried and evaluated for chemical composition and amino acid composition (Table 1) as well as peptide and amino acid contents by size-exclusion chromatography (Figure 2).

**Figure 2:**
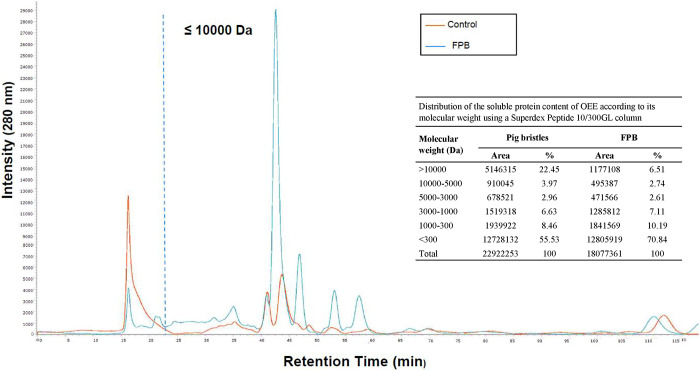
Chromatography profile of the soluble protein content of pig bristles and fermented pig bristles with *S. luteola* (FPB) according to its molecular weight using a Superdex Peptide 10/300 GL column. The table shows the distribution of the soluble protein content of the OEE according to its molecular weight using a Superdex Peptide 10/300GL column.

The main difference in chemical composition between pig bristles and FPB is the complete solubility of the latter as well as an increase in pH, which became alkaline (pH 8.2; Table 1).

Regarding the nitrogen content, an extraction of 16% nitrogen was achieved in the fermented extract. Like raw pig bristles, the amino acid content in FPB is quite varied, with glutamic acid standing out, reaching an even higher value than that in pig bristles (25.1 and 19.8%, respectively).

The content of peptides and amino acids larger than 10 kDa in the control soluble extract (22.45%) was higher than that in the fermented extract (6.5%), while the small protein/peptide (1–10 kDa) and small peptide/amino acid fractions (<1 kDa) increased from 77.55% (22.02 and 55.53%, respectively) in the control extract to 93.49% (22.65 and 70.84%) in the FBP extract (Figure 2).

### 3.5 Study on the Biostimulant Properties of the Soluble Fraction of Fermented Pig Bristles

#### 3.5.1 Soil Biostimulation Study

As shown in Figure 3, after applying FPB to soil, significant stimulation of dehydrogenase, phosphatase, and β-glucosidase activity was observed starting on day 7, reaching a peak between days 10 and 15, and returning to first-day values at the end of the experiment (day 30). The most prominent activity was dehydrogenase, which peaked at 10 days with a 45.5-fold increase compared to the SC control and a 3.2-fold increase compared to the soil sample treated with raw pig bristle (SPB).

**Figure 3:**
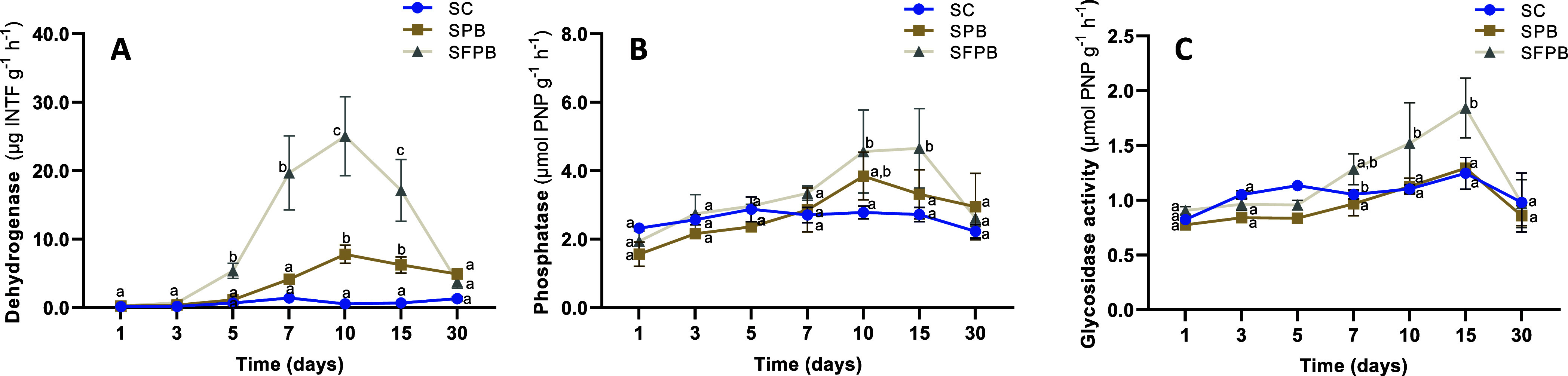
Enzyme activities in the control soil (SC) and experimental samples consisted of soil with the addition of raw pig bristle (SPB) or with the soluble fraction of fermented pig bristles from *S. luteola* (SFPB). Each product was evaluated at 1% w/w (dry matter). (A) Dehydrogenase activity; INTF, 2-*p*-iodo-3-nitrophenyl formazan. (B) Phosphatase activity; PNP, p-nitrophenol. (C) Glycosidase activity; PNP, p-nitrophenol. Values represent mean ± SD, *n* = 4. Points (mean ± SD) with the same letter(s) were not significantly different from each other (two-way ANOVA, HSD test, *P* < 0.05).

#### 3.5.2 Study in Plants

The physiological state of the plants was determined through various photosynthetic parameters such as A_N_, PhiPSII, and ETR as well as DF. After O_3_ exposure, *A*_N_, PhiPSII, and ETR were significantly affected (Figure 4A,B), with a notable decrease in *A*_N_, which dropped 7.2-fold compared to the control.

**Figure 4:**
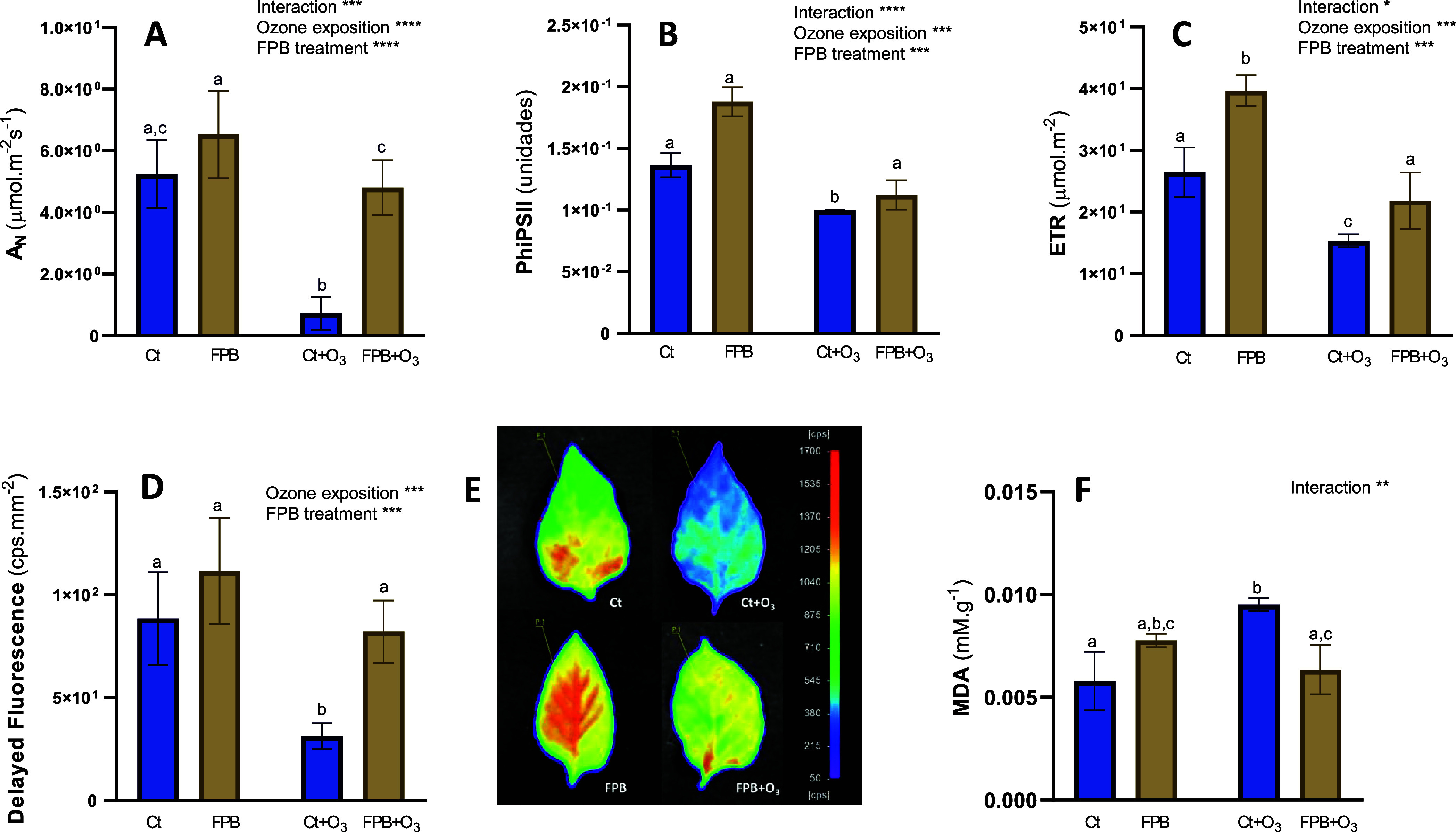
Biostimulant capacity of FPB in plants. Physiological parameters: (A) net photosynthetic rate (*A*_N_); (B) effective quantum yield of PSII (PhiPSII); (C) ETR and (D) delayed fluorescence (counts per second-cps) in leaves of pepper plants in response to ozone (O_3_) (100 ppb) under a treatment without and with soluble fermented extract (FPB). (E) Photographs taken by the plant imaging system NightShade LB 985. The color scale mirrors the detected cp’s of delayed fluorescence emission in leaves. (F) MDA concentration in the different groups. Ct, control plants sprayed with distilled water; Ct + O_3_, control plants exposed to ozone (100 ppb O_3_ for 6 h); FPB, plants sprayed with an aqueous solution of FPB at 1% (w/v); and FPB + O_3_, plants sprayed with FPB and exposed to ozone (100 ppb O_3_ for 6 h). Values represent mean ± SD, *n* = 5. Different letters indicate means that are significantly different from each other (two-way ANOVA, O_3_ exposition × FPB treatment; HSD test, *P* < 0.05). O_3_ exposition and FPB treatment in the corner of the panel indicate main or interaction significant effects (*P < 0.05; **P < 0.01; ***P < 0.0005; ****P < 0.0001).

Plants treated with FPB did not show significant effects on A_N_ or PhiPSII, and interestingly, in both groups, FPB treatment reversed the ozone-induced decrease, with the leaves of the FPB + O_3_ group showing no significant difference compared to the control group.

Regarding ETR, FPB treatment also restored the ozone-induced values to control levels, but this parameter appeared to be affected by FPB treatment, inducing a 1.5-fold increase in ETR.

DF was also measured (Figure 4D,E). DF is closely linked to photosynthesis reactions and has been used as a direct indicator of the chlorophyll content.^41^ O_3_ exposition clearly produced a loss of DF signals (2.8-fold decrease; Figure 4D), which was also restored to the control value by FPB treatment.

Finally, to analyze oxidative stress in plants following ozone exposure, antioxidant enzyme activities and MDA levels were measured. The ozone treatment induced a significant increase in the antioxidant activity of plants: CAT activity increased more than 2-fold (Figure 5A), SOD rose approximately 2.4-fold (Figure 5B), GPX activity tripled (Figure 5C), and APX activity also nearly tripled (Figure 5D), confirming an intense oxidative stress response. In contrast, the application of hair hydrolysate FBP led to a general reduction in enzyme activity: CAT decreased by approximately 1.5-fold, SOD dropped by about 1.6-fold, GPX showed a slight reduction (around 1.1-fold), and APX declined between 1.2- and 1.5-fold compared to the control, suggesting a protective or stress-alleviating effect. When ozone was combined with FPB, a general decrease in the antioxidant response was observed in comparison to that of ozone alone: CAT decreased by approximately 1.6-fold, SOD was reduced by 1.5-fold, GPX decreased around 2-fold, and APX dropped up to 2-fold.

**Figure 5:**
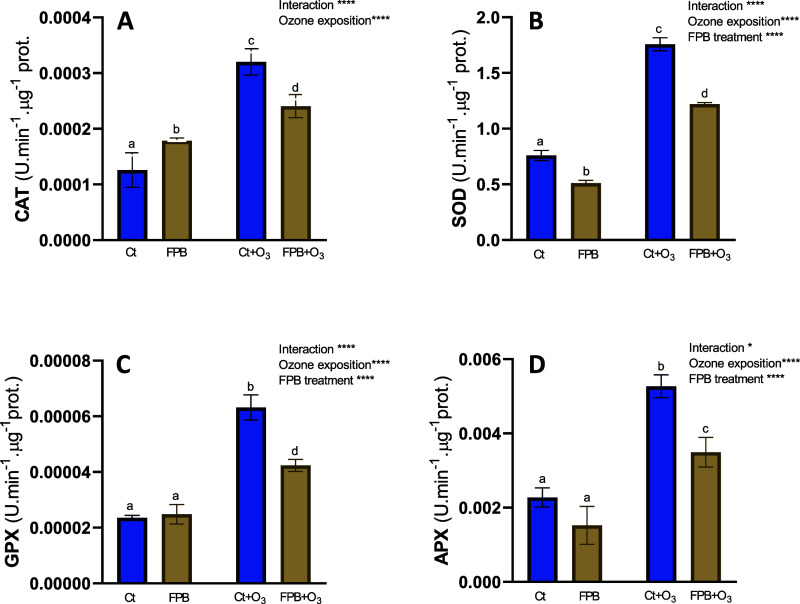
Antioxidant enzyme activities in leaves of pepper plants in response to ozone (O_3_) (100 ppb) under treatment without and with soluble fermented extract (FPB). (A) CAT, (B) SOD, (C) GPX, and (D) APX. Ct, control plants sprayed with distilled water; Ct + O_3_, control plants exposed to ozone (100 ppb O_3_ for 6 h); FPB, plants sprayed with an aqueous solution of FPB at 1% (w/v); and FPB + O_3_, plants sprayed with FPB and exposed to ozone (100 ppb O_3_ for 6 h). Values represent mean ± SD, *n* = 4. Different letters indicate means that are significantly different from each other (two-way ANOVA, O_3_ exposition × FPB treatment; HSD test, *P* < 0.05). O_3_ exposition and FPB treatment in the corner of the panel indicate main or interaction significant effects (*P < 0.05; **P < 0.01; ***P < 0.0005; ****P < 0.0001).

MDA is produced during lipid peroxidation and is commonly used as a marker for oxidative stress in plants. A lower MDA content indicates reduced oxidative damage and is considered a sign of more effective stress tolerance in plants.^42^ As shown in Figure 4F, ozone exposure caused a 1.6-fold increase in MDA, which was prevented when the plants were foliar-sprayed with FPB.

These findings suggest that hair-derived hydrolysates mitigate oxidative stress, likely due to the presence of peptides and amino acids with direct antioxidant properties and modulatory effects on cellular metabolism.

## 4 Discussion

This study demonstrates that the fermentation of pig bristles with bacteria isolated from the raw material itself produces a highly soluble extract rich in protein hydrolysates (PHs), which could protect plants against abiotic stress such as ozone exposure.

Keratinous waste from slaughterhouses, such as poultry feathers, pig bristles, and other similar materials, poses environmental challenges due to its low biodegradability. These materials are rich in keratin, a fibrous structural protein that is highly resistant to microbial degradation because of its tightly packed structure and the presence of disulfide bonds. Nonetheless, when processed with suitable enzymatic technologies, these byproducts could become a significant nitrogen source for enhancing soil biostimulation.^15^ In this context, in the present work, we have studied the possibility of obtaining a soluble extract rich in bioactives with biostimulant properties from pig bristles. For that, pig bristles were fermented with bacteria isolated from the raw material itself, identified as *B. licheniformis*, *S. luteola,* and *B. fordii* (Table 2). Based on the best results in PGP properties, different enzymatic activities (Table 3), and solubility (Figure 1A) and protease activity (Figure 1B) of the fermented extract, the fermentation process was carried out with *S. luteola*. The fermentation process increased the pH due to the release of ammonia during the biodegradation to peptides and amino acids.^43^

The insoluble fraction of fermented pig bristles obtained from *S. luteola*, rich in bacteria biomass (10^10^ ufc/g), shows potential as a biofertilizer due to its PGP traits (Table 3). FPB, the water-soluble extract, is rich in PHs, showing a higher content in small protein/peptide (1–10 kDa) and small peptide/amino acid fractions (<1 kDa) than the control extract (Figure 2). Interestingly, PHs, which are produced from the enzymatic hydrolysis of protein substrates into low-molecular-weight peptides and free amino acids, exhibit a range of biostimulant properties and are therefore categorized as biostimulants.^18^ When applied to soils, PHs have been shown to indirectly affect plant growth and nutrition by increasing nutrient availability and enhancing root absorption. This, in turn, promotes microbial activity and biomass in the soil, boosts soil respiration, and improves overall soil fertility.^18,44^

Like raw pig bristles, in FPB, the distribution of amino acids is quite varied, with glutamic acid standing out, reaching an even higher content after fermentation (25.1 vs 19.8% of the total; Table 1). Interestingly, it has been described that l-Glu is mainly involved in the defense of abiotic stress by plants,^45,46^ thus making keratins an interesting source of obtaining this amino acid.

Considering that soil enzyme activities can serve as indicators of soil quality,^17^ the current findings demonstrate that treating soil with pig bristles—especially after fermentation—enhanced soil fertility. This improvement is reflected in the induction of key metabolic enzymes, such as dehydrogenase, phosphatase, and glycosidase (Figure 3A–C). Notably, the activity of dehydrogenase increased significantly when the soil was treated with fermented FPB.

This enhanced enzymatic activity likely results from the action of proteases, breaking down keratins and soil proteins into more bioavailable compounds, increasing nitrogen availability in the soil. Interestingly, the dehydrogenase activity profile was similar to that observed in previous studies where peptide- and amino acid-rich compounds were added to the soil.^9,47^ However, stimulation was unexpected under conditions where raw keratins (SPB) were applied alone.

One plausible explanation for the high induction observed in soil after application of SFP could be the biostimulation of soil by increased nitrogen availability. This is likely due to the degradation of proteins within the soil organic matter by *S. luteola* from the fermented product, with enzyme activity decreasing as the organic matter becomes depleted. In the case of soil treated with pig bristles alone, the increase in dehydrogenase activity may result from the stimulation of specific proteolytic microorganisms, such as *S. luteola*, as well as other microorganisms indirectly benefiting from the enhanced nitrogen availability.

Regarding phosphatase and glycosidase activities, a slight significant increase was not detected until 10 days following the SFB treatment, which may be linked to the availability of phosphorus and carbon sources in the soil. Specifically, phosphatase activity is crucial for converting complex and sometimes unavailable forms of organic phosphorus into accessible phosphate as organisms can only assimilate dissolved phosphate. Thus, the production of phosphatase is influenced by a combination of phosphorus demand from plants and microbes, the presence of available organic phosphorus substrates, and phosphorus limitations in the soil.^48^ Meanwhile, glycosidase activity facilitates the hydrolysis of glycosidic bonds at terminal nonreducing residues in β-d-glucosides and oligosaccharides, leading to the release of glucose.^49^

One of the key benefits of biostimulants is their ability to induce stress tolerance in plants.^18^ To evaluate the biostimulatory capacity of FPB, we selected a model of abiotic stress induced by ozone exposure. The damage caused by ozone in living organisms arises from its high oxidizing power. Upon entry into the plant, ozone is degraded into reactive oxygen species (ROS) in the apoplastic space, potentially leading to oxidative stress. This stress causes direct or indirect ROS-mediated damage to various cellular components, including lipid peroxidation in membranes, protein denaturation, carbohydrate oxidation, and pigment degradation. The adverse effects of ozone on plants include a reduction in photosynthesis, increased water loss, and the appearance of chlorotic and necrotic spots on leaves.^50^

As expected, ozone exposure in pepper plants resulted in a decline in the photosynthetic parameters studied (Figure 4), with a particularly notable reduction in *A*_N_ (Figure 4A). This decline was partially reversed in plants treated with FPB. Similarly, the ozone-induced decrease in DF (Figure 4D,E), used as a direct indicator of the chlorophyll content, was mitigated by FPB treatment.

The keratinous nature of pig bristles, the primary raw material for FPB, results in a fermented extract rich in PHs, containing small peptides and amino acids, produced through enzymatic hydrolysis. Interestingly, PHs exhibit diverse antioxidant and free radical scavenging activities.^51^ Moreover, PHs have demonstrated the ability to enhance antioxidant mechanisms in plants.^52^ This antioxidant capacity likely explains the observed protection against ozone-induced oxidative damage, as supported by the antioxidant enzymes and MDA data. These findings suggest that the impairment of photosynthetic parameters is primarily due to oxidative stress, and the mitigation of this stress accounts for the observed recovery in photosynthetic function.

This stress-protective capacity aligns with the biostimulant properties of the PHs. PHs have emerged as a promising strategy in agriculture to enhance both plant growth and stress tolerance.^53^ As biostimulants, PHs have direct effects on plants, including modulating nitrogen uptake and assimilation, influencing signaling pathways in roots, and regulating enzymes involved in these processes.^18^ PHs also exhibit hormonal activity similar to auxin and gibberellin^54^ and producing antioxidant activity.^51,52^

Another significant component of FPB contributing to stress protection is its high glutamic acid content (Table 1). l-Glu plays a crucial role in plant growth and development.^55^ As highlighted earlier, l-Glu is a key player in plant defense mechanisms against abiotic stress. During stress conditions, l-Glu supports plant adaptation to environmental challenges, such as soil salinity, extreme temperatures, and water imbalances, whether caused by scarcity or excess.^56–58^

Therefore, the present results suggest that the composition of FPB makes it an excellent candidate for agricultural applications. We acknowledge that while this study focused on physiological and enzymatic parameters, as well as the characterization of PGP traits, a more comprehensive understanding of FPB’s mode of action will require the integration of molecular analyses. Future research should integrate assessments of stress-responsive gene expression and hormonal pathways—for example, through transcriptomic (RNA-seq) studies and field trials—to elucidate how FPB confers stress tolerance at the cellular and molecular levels.

The keratinous nature of pig bristles makes them a highly valuable waste material with significant potential for bioconversion into biostimulants rich in bioavailable nitrogen, peptides, and amino acids. In this study, we propose a method for valorizing pig bristles through fermentation employing specific keratolytic bacteria specially adapted and directly isolated from waste material. The resulting product, enriched with peptides and amino acids, is particularly well-suited as a biostimulant, a quality demonstrated in both soil experiments and its efficacy in enhancing plant protection against oxidative stress.

Our work contributes to ongoing efforts to minimize industrial waste while producing agronomic high-value products, presenting a novel approach with promising applications in the circular economy.

Nevertheless, the commercial implementation of pig bristle hydrolysates as biofertilizers faces key challenges, including regulatory requirements for biological safety, heavy metal content control, and agronomic efficacy validation; economic constraints due to high production, stabilization, and logistics costs, which affect competitiveness relative to synthetic fertilizers; and environmental concerns such as nitrogen leaching and soil metal accumulation. Addressing these challenges will require further research to ensure sustainable and viable agricultural use.
